# Aorto-Esophageal Fistula Caused by Vascular Malformation: A Case Description and an Analysis of the Literature

**DOI:** 10.3390/jcdd12070262

**Published:** 2025-07-08

**Authors:** Wenzhao Zhang, Xu Hu, Jianqun Yu

**Affiliations:** 1 Department of Radiology, West China Hospital, Sichuan University, Chengdu 610041, China; 2 Department of Emergency, West China Hospital, Sichuan University, Chengdu 610041, China

**Keywords:** aorto-esophageal fistula, computed tomography angiography, surgery

## Abstract

Aorto-esophageal fistula (AEF) is a condition with an extremely high mortality rate that often causes massive gastrointestinal bleeding, commonly resulting from esophageal perforation due to foreign bodies or aortic aneurysmal malformations. This case report introduces an elderly male patient who experienced hematemesis for longer than 16 h without obvious cause. The patient did not receive relief from endoscopic compression hemostasis. Through computed tomography angiography (CTA), a tortuous and thickened vessel was found in the descending aorta of the patient, which entered the esophagus. The diagnosis was AEF caused by vascular malformation. which has not been previously documented in the literature.

## 1. Introduction

Aorto-esophageal (AEF) is a rare and potentially fatal condition. The first confirmed case was reported in 1818, and successful treatment of AEF was not achieved until 1980 [1]. Due to diagnostic challenges and high misdiagnosis rates, AEF often leads to life-threatening gastrointestinal bleeding with a poor prognosis. Early recognition, accurate diagnosis, and prompt intervention are critical for improving patient outcomes. This article presents a rare case of primary AEF caused by vascular malformation, accompanied by a literature review. The mortality rate of AEF is extremely high. Early diagnosis and surgical treatment are of great help to the survival and prognosis of patients.

## 2. Case Report

A 60-year-old male was admitted to Shuangliu First People’s Hospital in Chengdu, Sichuan Province, with a chief complaint of hematemesis for longer than 16 h without obvious cause. The patient had no infection or trauma and had not swallowed any hard foreign objects such as pig bones, duck bones, metals, etc. Endoscopy revealed massive fresh blood and clots in the esophagus, with a jet-like bleeding site located 30 cm from the incisors. Hemostatic clips (10 in total) were deployed but failed to control the hemorrhage, which continued to gush through the gaps between the clips. A Sengstaken–Blakemore tube was then placed for tamponade, but the gastric cavity remained filled with blood, obstructing further visualization. During the procedure, the patient experienced cardiac arrest for approximately 20 min. After resuscitation, the patient was transferred to the West China Hospital of Sichuan University for further management.

**Figure 1:**
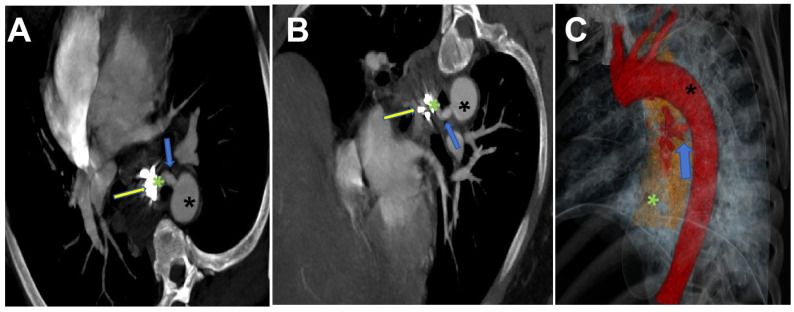
Axial MIP reconstruction (**A**), oblique coronal MIP reconstruction (**B**), and volume-rendered (VR) reconstruction (**C**) demonstrate a branching vessel (blue arrow) originating from the proximal descending aorta (black *) communicating with the esophagus (green*). High-density hemoclip artifacts are visualized within the esophageal lumen (yellow arrow). The VR image reveals significant blood pooling (blue arrow) in the esophageal cavity (green *).

**Figure 2:**
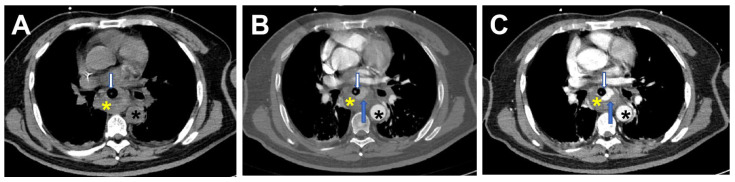
CTA examination: Non-contrast scan (**A**), arterial phase (**B**), and portal venous phase (**C**) images demonstrate a high-density enhancing shadow in the esophageal lumen (yellow *) on the non-contrast scan. During the arterial phase, a “crescent-shaped” higher-density shadow (blue arrow) is observed, which increases over time (**C**), suggesting active bleeding within the esophagus. A triple-lumen two-balloon tube is seen in the esophageal lumen (white arrow). Descending thoracic aorta (black *).

Chest computed tomography angiography (CTA) demonstrated discontinuity in the mid-esophageal wall, with swelling of the surrounding soft tissue swelling, effusion, and gas accumulation. A tortuous vascular structure (diameter: 0.5 cm) originating from the descending thoracic aorta extended into the esophageal lumen (Figure 1). The esophageal cavity also exhibited a high-density shadow, which progressively increased, suggesting active bleeding (Figure 2). Laboratory tests revealed severe anemia (RBC: 1.83 × 10^12^/L, Hb: 58 g/L, Hct: 0.18 L/L), thrombocytopenia (91 × 10^9^/L), and hypoalbuminemia (26.0 g/L).

**Figure 3:**
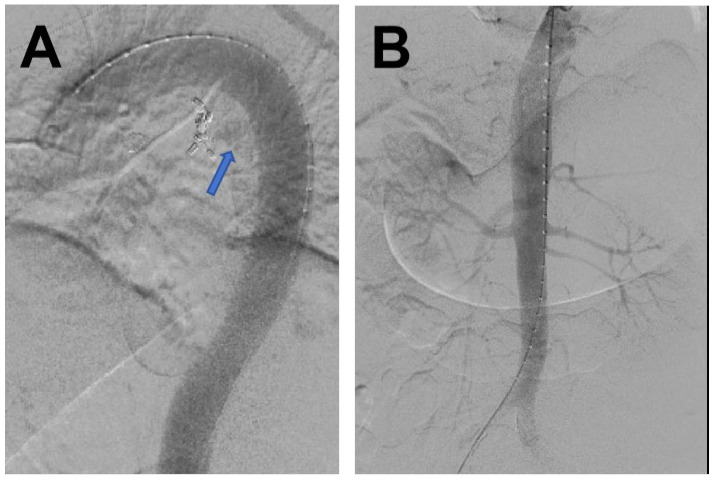
The descending aorta demonstrates patency throughout its course. At the proximal segment, a branching vessel (blue arrow) is observed communicating with contrast extravasation into the adjacent esophageal lumen (Panel **A**). The descending aorta and its major branches remain fully patent without evidence of obstruction or flow limitation (Panel **B**).

**Figure 4:**
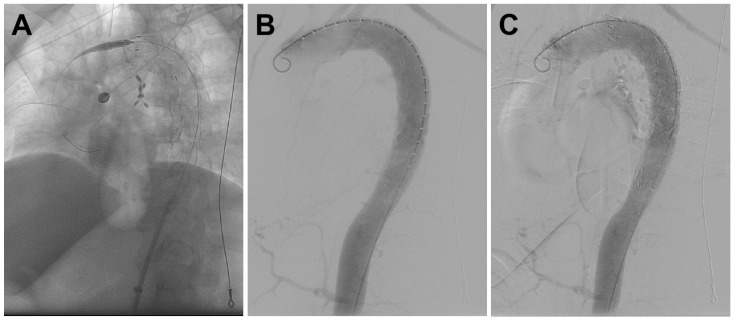
(**A**–**C**) Following endovascular stent deployment over the guidewire, follow-up angiography demonstrated Patent stent lumen with unimpeded blood flow and the absence of contrast extravasation.

The patient underwent emergency thoracobdominal aortography+thoracic aortic stent placement surgery in the hybrid room under general anesthesia. Pigtail catheter angiography revealed a patent descending aorta, with a proximal branch vessel communicating with the esophagus, showing ill-defined patchy contrast staining in the adjacent esophageal region, while the descending aorta and its branches remained patent (Figure 3). After exchanging for a super-stiff guidewire, a Medtronic VAMF3232C200TE stent graft (Medtronic Inc., Parkmore Business Park West, Galway, Ireland) was used. The stent was deployed with its proximal end distal to the origin of the left subclavian artery and its distal end in the lower thoracic aortic segment. Follow-up angiography demonstrated patent blood flow within the stent and no contrast extravasation (Figure 4), with no visualization of the previously noted descending aortic branch vessel.

The patient was postoperatively transferred to the intensive care unit for further management. Despite receiving packed red blood cell transfusions, fresh frozen plasma infusions, and intramuscular medications, the patient’s hemoglobin levels continued to decline progressively. No clinical improvement was achieved, and the patient was declared deceased 12 h after the surgical procedure.

## 3. Discussion and Conclusions

AEF is a rare disease that can cause massive gastrointestinal bleeding. Based on its etiology, it can be classified into primary AEF or secondary AEF. Primary AEF refers to cases in which esophageal foreign bodies, diseases (including aortic aneurysms, esophageal cancer, bronchogenic carcinoma, etc.), or thoracic trauma impair the aortic wall, with aortic rupture being the most common cause. Secondary AEF mostly occurs in patients with a history of aortic surgery or adjacent organ surgery [2]. The development of AEF is related to the anatomical structure of the esophagus and thoracic aorta. Foreign bodies and esophageal cancers frequently occur at the site where the esophagus passes behind the left main bronchus and crosses with the thoracic aorta (the second esophageal constriction), and this is also the most common site for AEF formation [3]. Additionally, infections or atherosclerotic changes in the aortic wall at this location can lead to localized arterial dissection, aneurysms, or pseudoaneurysms. Combined with the reduced elasticity of atherosclerotic vessel walls and vascular compression on the esophagus, this may result in ischemic necrosis of the esophageal wall, ultimately forming an aorto-esophageal fistula [4].

The typical clinical manifestation of AEF is characterized by Chiari’s triad: retrosternal pain, sentinel bleeding, and intermittent massive fatal hemorrhage [5]. Retrosternal pain may be caused by the expansion or penetration of arterial/esophageal lesions. The initial “sentinel bleeding” may present as intermittent or minor gastrointestinal bleeding, likely related to temporary hemostasis from blood clots blocking the fistula. As clots dissolve, dislodge, or become infected, or as continued bleeding increases pressure on the rupture site, larger ruptures may occur, leading to a fatal hemorrhage manifesting as massive hematemesis. However, some patients may experience fatal bleeding during their first episode [6,7,8].

AEF is challenging to diagnose, and few patients present with all three components of Chiari’s triad. Hollander et al. [2] reported that 59% of patients experienced chest pain and 65% had “sentinel bleeding,” while only 45% manifested the complete triad. Consequently, primary AEF is frequently misdiagnosed in clinical practice. Once massive bleeding occurs, mortality is extremely high, making early diagnosis crucial for a better prognosis. The literature indicates that endoscopy is the most sensitive and specific diagnostic method for AEF [9]. However, the role of endoscopy in diagnosing AEF remains controversial due to its complexity and risk of inducing fatal hemorrhage [10]. For patients with high suspicion of AEF, non-invasive methods like a CT scan or CTA may be preferable. The characteristic CTA findings in AEF typically include an incomplete aneurysm wall, contrast extravasation, and mediastinal free gas [5].

AEF has an extremely high mortality rate, and there are currently no reported cases of successful endoscopic hemostasis for AEF-induced massive gastrointestinal bleeding [11,12]. Surgery remains the primary treatment for AEFs, though there is no consensus on the optimal surgical approach [13,14,15,16]. Takeno et al. [17] analyzed the correlation between different surgical approaches and patient outcomes. Endovascular aortic repair is often preferred as the initial surgical option due to its minimal invasiveness and effective hemostasis. For an AEF caused by esophageal lesions, esophagectomy is the treatment of choice. Li et al. [18] found that conservative treatment of AEFs carries an extremely high mortality rate, with hemorrhagic shock, sepsis, and multiple organ failure being the main risk factors for death. The combination of aortic replacement and esophagectomy can significantly improve patient survival and prognosis.

The present patient presented with “hematemesis without obvious cause” and had no history of stent implantation, esophageal foreign bodies, ulcers, inflammation, or tumors. The absence of previous melena (“sentinel bleeding”) or Chiari’s triad made the case particularly misleading, leading to an initial misdiagnosis of conventional gastrointestinal bleeding. The referring hospital performed an endoscopy to investigate the bleeding source, revealing jet-like arterial bleeding in the esophageal lumen that was unresponsive to hemoclip placement or balloon tamponade, prompting the transfer to our institution. Our chest CTA revealed a tortuous, enlarged vessel originating from the proximal descending aorta and traversing into the esophageal lumen, with a discontinuous adjacent esophageal wall and surrounding free gas, confirming the diagnosis of AEF. This represents a rare case of a primary AEF caused by a vascular malformation, previously unreported in the literature [17]. CTA precisely identified the bleeding source, vascular anomaly location, and its relationship with adjacent structures, providing crucial guidance for surgical planning. The non-invasive nature of CTA also avoided the risk of provoking or exacerbating bleeding during diagnostic procedures. Unfortunately, the malformed vessel originating from the thoracic aorta exhibited a high flow volume and pressure, and even after aortic stent placement, bleeding from the ruptured anomalous vessel persisted. Combined with the patient’s massive blood loss prior to admission and brief cardiac arrest, and continuously decreasing postoperative hemoglobin levels, the patient rapidly succumbed despite resuscitation efforts.

Although AEF is clinically rare, its high mortality necessitates increased clinical awareness. Early diagnosis and prompt surgical intervention are critical for improving survival rates. Patients with gastrointestinal bleeding and risk factors for AEF (including a history of esophageal foreign bodies [retained or removed], esophageal cancer, esophageal stent placement, aortic dissection stent placement, or aortic aneurysms) should be carefully evaluated for possible AEF. A timely chest CT scan, aortic CTA, or even aortography should be performed for accurate diagnosis. Regardless of the etiology, early surgical intervention remains the best potential life-saving approach for AEF patients.

## Data Availability

The original contributions presented in this study are included in the article. Further inquiries can be directed to the corresponding author(s).
